# Real-Time On-Site Multielement Analysis of Environmental Waters with a Portable X-ray Fluorescence (pXRF) System

**DOI:** 10.1021/acs.analchem.2c01490

**Published:** 2022-08-16

**Authors:** Tommi E. Tiihonen, Tuomo J. Nissinen, Petri A. Turhanen, Jouko J. Vepsäläinen, Joakim Riikonen, Vesa-Pekka Lehto

**Affiliations:** † Department of Applied Physics, University of Eastern Finland, Yliopistonranta 1, FI-70211 Kuopio, Finland; ‡ 3AWater Oy, Microkatu 1, FI-70210 Kuopio, Finland; § School of Pharmacy, University of Eastern Finland, Yliopistonranta 1, FI-70211 Kuopio, Finland

## Abstract

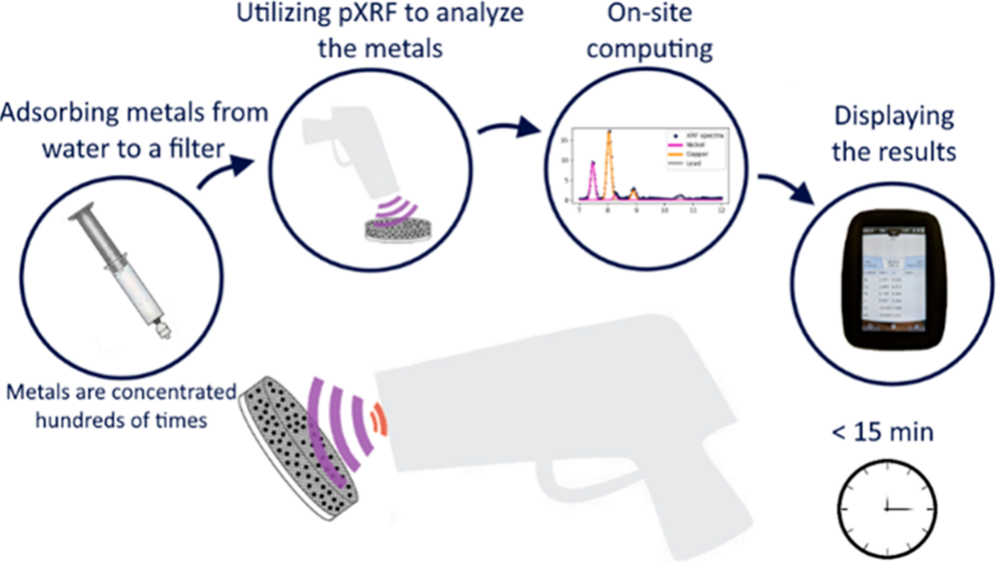

Strict regulations are in place to control the effluents of mining sites and other industries. Heavy metal contamination of aquatic systems caused by leakages is difficult to mitigate as it takes time to detect and localize the leak. Dynamic sampling would drastically reduce the time to locate leakages and allow faster actions to reduce the impact on the environment. The present study introduces a novel portable multielement water analysis system to simultaneously measure Mn, Ni, Cu, Zn, Pb, and U in water samples from natural sources within 15 min from the sampling. The metals are preconcentrated from a 10 mL water sample into a nanoporous filter based on bisphosphonate-modified thermally carbonized porous silicon. The metals can be conveniently analyzed from the filter with a portable XRF analyzer in field conditions. The system was empirically calibrated for a lake water matrix with neutral pH and low alkaline metal concentration. A strong correlation between the XRF intensities and the ICP-MS results was obtained in a concentration range from 50 to 10 000 μg/L. With a DPO-2000C XRF analyzer, the detection limits were 103, 86, 92, 35, 44, and 43 μg/L for Mn, Ni, Cu, Zn, Pb, and U, respectively. The corresponding values with X-MET8000 Expert Geo were 137, 46, 62, 38, 29, and 54. The system was successfully validated with simulated multielement lake water samples and piloted in field conditions. The system provides an efficient way to monitor metals in environmental waters in cases where quick on-site results are needed.

Water pollution caused by heavy metals is a significant global problem. The main sources of metal pollution are mining and manufacturing, fertilizer and pesticide use, rock weathering, and wastewater discharge. Processes in metal and mining industries use a lot of water that contains large amounts of heavy metals which need to be removed before releasing the water back to the environment.^1−4^ Even small concentrations of heavy metals, especially in dissolved form, can be very toxic to plants and organisms.^5,6^ Monitoring low metal concentrations with technologies currently available is complicated, and expensive laboratory techniques are still needed. Laboratory measurements require sampling on site, conditioning of the sample, fast shipping to the laboratory, and analysis with techniques such as inductively coupled plasma mass spectrometry (ICP-MS), optical emission spectrometry (ICP-OES), or atomic absorption spectroscopy (AAS).^7^ The cost of this chain is high due to shipping logistics, laboratory equipment and reagents, and labor costs of skilled personnel. It takes days to obtain the results, precluding any real-time information for fast decision making. Real-time data of the metal concentrations in water would be useful, for example, in managing industrial emissions and process waters and monitoring domestic water quality. A dynamic sampling plan, where the real-time data from previous water samples inform the choice of the next sampling point, would speed up finding sources of leakages and prevent further damage to the environment.^8^ Furthermore, emission control is of financial interest for industries as emissions exceeding the limits set by authorities result in damage to their reputation and high costs.

In recent years, different preconcentration procedures combined with portable X-ray fluorescence (pXRF) spectrometry to analyze metal concentrations in aqueous samples have been reported.^9−16^ Preconcentration improves the analytical detection limits of the XRF technology from a milligrams per liter (mg/L) to a micrograms per liter (μg/L) level to meet the environmental guidelines for dissolved metals. The analytical method to be utilized in field conditions needs to be simple with minimal sample preparation. Many of the preconcentration procedures, such as evaporation and precipitation with chelating agents, require time and chemicals that need to be handled carefully, hindering analysis in field conditions. Hagiwara et al. reported a method utilizing an anion-exchange disk to preconcentrate metals.^11^ The disks were analyzed with a pXRF, and the metal concentration of the initial water sample was calculated using empirical calibration. Although the system does not require an electrical outlet or use of toxic chemicals, it still requires the operator to prepare the sample disks for the XRF measurement by taping them on both sides with cellophane tape and drying the disks with a cordless hair iron. The pH of the water sample also needs to be adjusted. Other approaches to preconcentration found in the literature are listed in Table S1 of the Supporting Information.

In the present study, we introduce a novel multielement water analysis system to monitor low metal concentrations in environmental waters to address the challenges related to field-operated instrumentation. The method is based on a functional nanostructured hybrid material that concentrates cationic metals from low-concentration aqueous solutions into a solid state. This hybrid material comprises a nanoporous silicon matrix with a durable silicon carbide layer on its surface.^17,18^ Attached to the silicon carbide surface are bisphosphonate molecules that efficiently collect metal ions over a wide pH range (2–12).^18−20^ The hybrid material is fastened to a cellulose filter support with poly(acrylic acid) and carboxymethylcellulose sodium binders to create metal collecting filters (MCF). The binders were selected to enhance the adsorption of the metals.^21−23^ The water sample is pumped through the MCF where the metals are concentrating several hundreds of times. This allows metals to be directly measured from the MCF using a relatively inexpensive and insensitive pXRF. Though production of the adsorbent used in the MCFs is expensive when manufactured in small scale, the filters only require a small amount of it. The manufacturing cost was calculated to be 8 euros per filter with potential to scale up. With this system, the measurements can be performed on site in 15 min per sample. The advantages of the developed system are, in addition to fast on-site measurements and easy sample preparation, the wide concentration range and the simultaneous measurement of several metals.

## Experimental Section

### Adsorbent Production

The porous silicon (PSi) used in the MCFs was prepared from Si wafers (p++ type, ρ = 0.01–0.02 Ω cm, Okmetic Oy) by electrochemical etching. Etching was done at a current density of 30 mA/cm^2^ for 40 min, and a 1:1 solution of hydrofluoric acid (HF 38–40%, Merck) and ethanol (EtOH 99.5%, Altia Oyj) was used as the electrolyte. The etched PSi layer was detached from the wafer with a high-current pulse. PSi films were dried at 65 °C and milled (400 rpm, 4 min) with a planetary ball mill (Fritsch Pulverisette 7). The PSi particles were sieved into size fraction below 25 μm.

The PSi particles were surface treated to create a stable silicon carbide layer.^24^ One gram of PSi particles was submerged in a HF/EtOH (1:1) solution for 10 min and then dried at 65 °C for 40 min. The dry particles were moved into a quartz tube and flushed with 1 L/min N_2_ flow at RT for 30 min. The N_2_ flow was kept on for the rest of the process. After 30 min, 1 L/min C_2_H_2_ flow was added for 15 min before inserting the quartz tube into a 500 °C tube oven for 14 min 30 s. The C_2_H_2_ flow was cut off, and the tube was kept in the oven for another 30 s. The tube was cooled at RT for 30 min. C_2_H_2_ flow was resumed for 9 min 40 s. Twenty seconds after cutting off the C_2_H_2_ flow, the tube was inserted into an 820 °C tube oven for 10 min. Produced thermally carbonized PSi (TCPSi) powder was cooled to RT and kept under N_2_ atmosphere.

Bisphosphonate (BP, tetrakis(trimethylsilyl) 1-(trimethylsilyloxy)undec-10-ene-1,1-diylbisphosphonate) was synthesized using the method reported by Riikonen et al.^18^ A 0.5 g amount of BP molecules was mixed in 10 mL of mesitylene (99% extra pure, ACROS Organics) in a two-necked flask with one neck connected to a N_2_ inlet and the other to the cap of the quartz tube with a Teflon tube. The solution was degassed by bubbling with N_2_ for 40 min and injected into the quartz tube with 1 g of TCPSi particles inside. The quartz tube was sealed with a N_2_ atmosphere inside. The sample was incubated at 120 °C for 19 h. The mesitylene, unbound BP molecules, and protective trimethylsilyl groups were washed away with 200 mL of MeOH, and the BP-TCPSi sample was dried at 65 °C for 1 h. A reference TCPSi sample for calculating the BP content of the final product was produced in a similar manner except no BP was used.

### Metal Collecting Filter

The MCFs used in the pXRF system were produced by dispersing BP-TCPSi particles in deionized (DI) water with poly(acrylic acid) (PAA, MW 100 000, 35 wt % in H_2_O, Sigma-Aldrich) and carboxymethylcellulose sodium salt (CMC, η = 50–200 cP, in 4% H_2_O, Sigma-Aldrich) acting as binders to form a slurry. Good mechanical strength of the MCFs was achieved when the mass percent composition of the dry coating was 80% BP-TCPSi, 10% PAA, and 10% CMC. The viscosity of the slurry was suitable for the filter coating when the DI water amount was 3.15 mL per 1 g of BP-TCPSi.

The slurry was prepared by first mixing PAA in DI water. CMC was added, and the mixture was stirred for 1 h. BP-TCPSi particles were added when the CMC was dissolved, and the slurry was mixed for another 1 h. The slurry was spread on a filter paper (Whatman grade 3) with a film coater (TMAX-TM) set to thickness of 1200 μm. The filter paper was dried at RT for 30 min before putting into a 150 °C vacuum oven for 2 h for the binders to form a cross-linked structure that binds the particles together and to the filter paper.^25^ The filter sheet was cooled to RT and cut into round filters 13 mm in diameter. The finished filters had approximately 15 mg of BP-TCPSi each and a coating thickness of 330 μm.

### Material Characterization and Instrumentation

The size distribution of the BP-TCPSi particles was measured with laser diffraction (Mastersizer 2000, Malvern Instruments, UK) using EtOH as dispersant. The surface area, pore volume, and pore diameter of the BP-TCPSi particles and the cross-linked filter coating scraped off the support filter paper were measured with N_2_ gas sorption (Micromeritics Tristar II 3020). The surface area was calculated from the measured adsorption isotherms using the Brunauer–Emmett–Teller (BET) method. The single-point pore volume was determined from the adsorption branch at *p*/*p*° = 0.98 and the pore size distribution from the desorption branch using the Barrett–Joyner–Halenda (BJH) method. The BP content (wt %) in the BP-TCPSi particles was measured with a thermogravimetric analyzer (TGA, NETZSCH TG 209 F1 Libra) by comparing the mass loss of the BP-TCPSi and unfunctionalized TCPSi particles.

### Multielement Water Analysis System

The MCFs were loaded in 13 mm Swinnex filter holders (SX0001300, Merck), and a battery-operated syringe pump (NE-1000, New Era Pump Systems, Inc.) with 20 mL plastic syringes was utilized to pump liquid through the MCFs. The MCFs were first primed by pumping 5 mL of 1 M H_2_SO_4_ through the MCF with a flow rate of 0.2 mL/min. The acid was washed away with 5 mL of DI water with a flow rate of 1 mL/min. After priming, 10 mL of metal-contaminated water was pumped with a flow rate of 1 mL/min first through a 0.45 μm nylon membrane filter (VWR) to collect the solid material and then through the MCF as one process. The prefilter was chosen to be the same type as what the commercial laboratories use when sampling water on-site. This was done to exclude the possibility of the prefilter affecting the results. After filtration, excess water was pushed out of the filter holder with air. The holder was disassembled, and the adsorbed metals were measured from the wet MCF with a pXRF.

**Figure 1:**
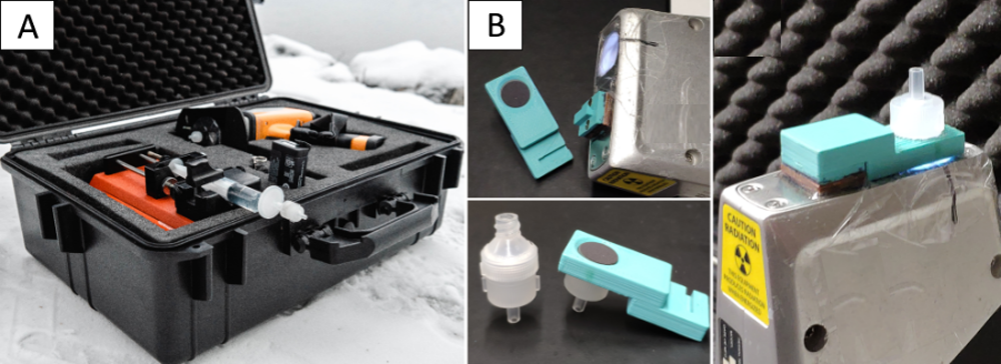
(A) pXRF water analysis system. (B) MCF attached on the snout of the pXRF analyzer with a 3D-printed adapter.

XRF analyses were performed using DPO-2000C and X-MET8000 Expert Geo XRF analyzers. DPO-2000C was used with the integrated Soil method which has three set of parameters optimized for lighter and heavier elements. The first set of parameters (*V* = 40 kV, *A* = 80 μA, filter 3) was used to measure U. The second set (*V* = 40 kV, *A* = 83 μA, filter 1) was used to measure Ni, Cu, Zn, and Pb. The third set (*V* = 15 kV, *A* = 125 μA, filter 5) was used to measure Mn. The irradiation time for each parameter was 40 s, making the total measurement time 2 min. XRF spectra measured with these parameters are shown in Figure S1 of the Supporting Information. With X-MET8000, a custom method was created using filter 6 with the voltage set to 45 kV and current to 30 μA. The irradiation time was 2 min. The MCF was attached on the snout of the pXRF with a 3D-printed bracket (Figure 1). This enabled accurate orientation and a constant distance of the MCF in relation to the exposure window of the instrument. The XRF spectra were exported to a laptop, and elemental concentrations of the water were calculated using empirical calibrations prepared for the analyzer.

### Empirical Calibration

A 10 mL amount of single-metal-spiked lake water samples was filtrated through the MCFs using the flow-through setup (Figure 1) as follows. Water was first prefiltered through a 0.45 μm nylon membrane filter to separate suspended solids. The water was then spiked with Mn, Ni, Cu, Zn, Pb, and U metal standards (1000 mg/L) of MnCl_2_, NiCl_2_, CuCl_2_, ZnCl_2_, Pb(NO_3_)_2_ (Titrisol, Merck), and U_3_O_8_ (AA66N-5, AccuStandard). The spiked metal concentrations ranged from 50 to 10 000 μg/L. After metal spiking, the pH was adjusted back to the original value of 7 using NaOH. The samples were pumped through the MCFs at a 1 mL/min flow rate. After filtration, the holder was disassembled, and the metals on the MCF were measured with the pXRF. The metal concentrations of the initial and filtrated water samples were measured with ICP-MS (Nexion 350D, PerkinElmer), and the adsorption efficiencies of the metals were calculated from the change in concentration. DI water was filtrated as a blank sample. The metal content in the MCF was determined from the characteristic emission lines of the XRF spectra (Table S2, Supporting Information). The calibration curves were created by plotting the XRF counts as a function of the initial metal concentrations of the water samples measured with ICP-MS and fitting a regression line on the data points.

### Effect of Water Matrix

To study the effects of pH and the amount of Mg and Na in the water on the ability of the MCF to effectively capture the metals, metal solutions containing all six calibrated metals were prepared at pH 5, 6, 7, and 8.5 since that is the usual pH range of environmental water samples. The pH was adjusted with NaOH. The effect of Mg was tested with three spiking levels of 6.0, 12.4, and 25.0 mg/L and Na with two spiking levels of 13.6 and 27.4 mg/L. The results were compared at each pH to the results from the lake water sample with the original concentrations of 2.5 mg/L of Mg and 7.2 mg/L of Na. To make sure the metals were detectable even in the worst-case scenario, the spiked metal concentrations were chosen to be relatively high between 800 and 1000 μg/L for Mn, Ni, Cu, and Zn. For Pb and U, the concentrations were around 400 μg/L.

### System Validation

The performance of the pXRF system was verified with six spiked multielement lake water samples prepared and measured with ICP-MS by an external laboratory. The pH of the water samples was 7. ICP-MS results were compared to the results given by the system. The system was also used in the field near two old industrial sites to measure the metal concentrations from a total of 16 groundwater and surface water samples. The water samples were analyzed on site with the pXRF system and sampled for ICP-MS analysis.

## Results and Discussion

### Characterization of the Materials

The median size of the BP-TCPSi particles was 14.9 ± 0.2 μm (Figure S2, Supporting Information), and the BP content according to TGA was 1.25 ± 0.04% w/w (Figure S3, Supporting Information). The BET surface areas, pore diameters, and pore volumes of the BP-TCPSi particles and the filter coating containing binders are shown in Table 1. The N_2_ sorption isotherms are presented in Figure S4 of the Supporting Information. The BP-TCPSi particles had a specific surface area of 234 m^2^/g before addition of binders. The binders reduced the surface area by 46% and the pore volume by 38%. The reduction is partially due to lower BP-TCPSi content (80%), but some pores of the BP-TCPSi were also blocked by the binders.

**Table 1:** BET Surface Area, Average Pore Diameter, and Pore Volume of BP-TCPSi Particles and Filter Coating (mean ± σ, *n* = 3) Measured with N_2_ Sorption

material	surface area (m^2^/g)	pore diameter (nm)	pore volume (cm^3^/g)
BP-TCPSi	234 ± 2	10.0 ± 0.3	0.61 ± 0.01
filter coating	126 ± 2	9.9 ± 0.2	0.38 ± 0.01

### Regression Analysis of the Empirical Calibration Data

Calibration curves were determined for Mn, Ni, Cu, Zn, Pb, and U. The calibration data was obtained by filtrating 10 mL of lake water samples spiked with one metal through the MCFs. The moist MCFs were measured with the pXRF after filtration as drying is complicated to be performed in field conditions. Nevertheless, it did not affect the measured XRF intensities significantly whether the MCFs were measured wet or dry (Figure S5, Supporting Information). Calibration curves for the six metals were linear regression equations (Figures S6 and S7, Supporting Information). The adsorption efficiency of the metals change depending on the spiking level (Figure S8, Supporting Information). For Mn, Ni, and Zn, the efficiencies decrease when the initial metal concentration of the water sample increases above 1 mg/L. For Mn, the efficiency decreases from 79% at 1 mg/L to 44% at 10 mg/L. For Ni, the drop in efficiency is from 65% to 46% and for Zn from 87% to 59%. In the case of Ni, the adsorption efficiency also decreased when the initial metal concentration decreased below 1 mg/L, falling to 46% at 0.06 mg/L. For Cu, Pb, and U, the efficiency improves as the metal concentration increases. Cu adsorption increases from 70% to 90%, Pb from 84% to 98%, and U from 83% to 94%. Nevertheless, a linear fit was found to be working well.

The limit of detection (LOD) of each metal was estimated by calculating the following XRF intensity (*I*_XRF_)
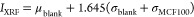
where *μ*_blank_ is the mean and *σ*_blank_ is the standard deviation of the XRF counts from 10 repeat measurements of blank MCFs. *σ*_MCF__100_ is the standard deviation between XRF counts measured from MCFs used for 0.1 mg/L calibration data points (*n* = 3). The calculated XRF intensities were inserted in the calibration equations to acquire the LODs for the system (Table 2).^26^ The LODs are on a level suitable for analysis of polluted waters with a pH close to neutral and low alkaline metal concentration. For the system to be suitable for measurement of potable water, the detection limits should be lowered considerably.

**Table 2:** LODs of the pXRF System (in μg/L)^a^

XRF device	Mn	Ni	Cu	Zn	Pb	U
DPO-2000C	103	86	92	35	44	43
X-MET8000	137	46	62	38	29	54

a Valid for a lake water matrix with a Mg concentration of 2.5 mg/L and pH 7.

### Effect of Water Matrix

The pXRF system was tested with water samples containing different Mg and Na levels with pH values of 5, 6, 7, and 8.5 to determine how these parameters affect the performance of the system. Mn, Ni, and Zn suffered the most. At pH 7, the comparison with the control sample showed that the measured concentration of the three metals dropped 25.6–54.3% when 6.0–25.0 mg/L Mg was added and 12.2–25.0% when 13.6–27.4 mg/L Na was added. In the case of Cu, Pb, and U, the matrix effect was less severe and the measured concentrations were within the margin of error.

The pXRF system measured a 34.4–45.5% decrease in the control sample concentrations of Mn, Ni, and Zn at pH 5 and a 21.7–29.0% decrease at pH 6 when compared to pH 7. The Pb and U concentrations increased by 23.8–36.3% at pH 5. The Cu concentrations were within the margin of error. At pH 8.5, the Mn concentration increased by 14.8%. pH 5 in combination with the highest added Mg showed the largest changes in the measured concentrations where Mn, Ni, and Zn decreased 63.7–75.9%. The measured metal concentrations and adsorption efficiencies are shown in Figures S9–S14 of the Supporting Information.

### Field Measurements and Simulated Samples

**Figure 2:**
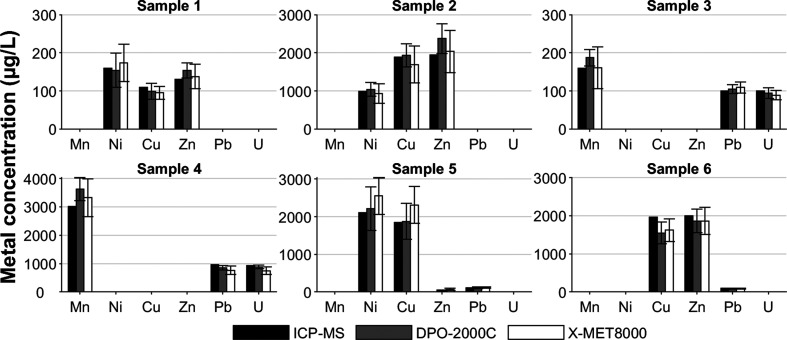
Multielement spiked lake water samples (*n* = 5, *n* = 4 for sample 5) measured using the pXRF water analysis system utilizing the empirical calibrations prepared with the water samples of comparable lake water matrix.

Six metal-spiked lake water samples were provided by an external laboratory and measured with the pXRF system. The average results were within 25% of the ICP-MS results (Figure 2). The standard deviation of the pXRF system measurements was between 6.3% and 34.2%. This could be caused by microcracks on the MCF that can occur when the filter holder is assembled. Cracks can cause the water to pass the filter without proper contact with the adsorbent.

**Figure 3:**
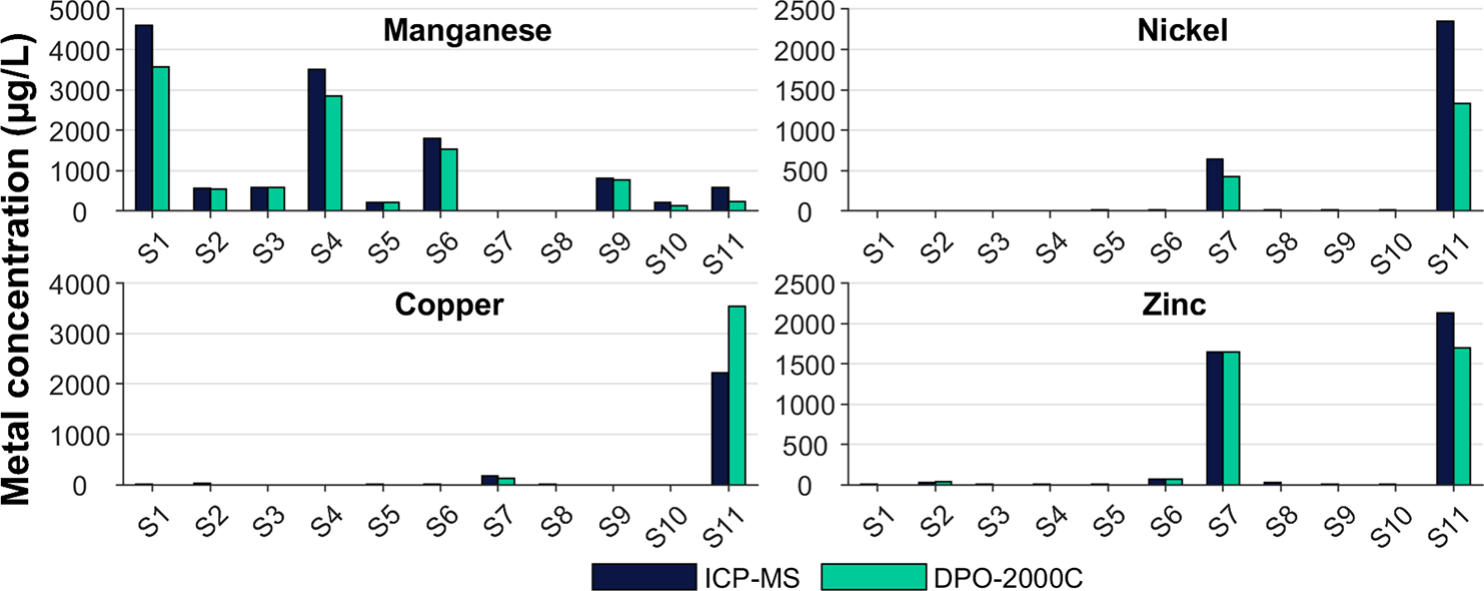
Total of 16 groundwater and surface water samples were measured with the pXRF water analysis system in a field pilot near two old industrial sites (*n* = 1). Only the results from water samples containing metals above the detection limits are shown.

A total of 16 groundwater and surface water samples were analyzed in field conditions with the pXRF system. The measured metal concentrations were slightly underestimated by the pXRF system when compared with the ICP-MS results (Figure 3). Higher concentrations of alkaline metals, such as Mg, were found in the analyzed waters when compared to the water samples used in the calibration of the system. Mg concentrations were up to 18 mg/L compared to a value of 2.5 mg/L in the calibration samples. The matrix effect tests show that the analyses of Mn, Ni, and Zn are affected by the pH and Mg amount in the water. It should be noted that each water sample was measured with the system only once. The temperature, electrical conductivity, pH, and metal concentrations of the water samples are listed in Table S3 of the Supporting Information.

## Conclusions

An on-site, multielement water analysis system combining a portable XRF spectrometer and a nanostructured metal collecting filter was developed. The system can measure dissolved Mn, Ni, Cu, Zn, Pb, and U in polluted environmental waters with pH close to neutral within 15 min from sampling with minimal sample preparation. The performance of the system was validated with simulated metal-contaminated water samples and piloted in field conditions with promising results. The system provides an efficient way to monitor metals in environmental waters in cases where quick on-site results are needed.

The developed system was prone to adverse water matrix effects caused by pH and high Mg levels when measuring Mn, Ni, and Zn. If any preliminary data about the alkaline metal levels is not available, this shortcoming is severe and would need site-specific calibration. In the case of Cu, Pb, and U, the matrix effect caused by the pH and Mg levels was minor.

## Supporting Information Available

Different approaches to preconcentration of metals for XRF measurement; XRF spectra of a MCF with adsorbed metals compared to blank measurement; XRF emission lines used for analysis; size distribution of the BP-TCPSi particles; TGA data with BP content of BP-TCPSi particles; N_2_ sorption isotherms measured with BP-TCPSi and the filter coating; XRF intensities with dry and wet MCF; empirical calibration data; adsorption efficiencies of the empirical calibration samples; matrix effect test data; supplementary data of the field measurement water samples (temperature, electric conductivity, pH) (PDF)
