# Supplementary material for: Real-Time On-Site Multielement Analysis of Environmental Waters with a Portable X-ray Fluorescence (pXRF) System
Source: Anal Chem. 2022 Aug 16;94(34):11739–44. doi: 10.1021/acs.analchem.2c01490 (PMC9434549; doi:10.1021/acs.analchem.2c01490)
Supplement: Supplementary file 1 — ac2c01490_si_001.pdf [file ac2c01490_si_001.pdf]

Supporting information

Real-time on-site multi-element analysis of environmental waters with portable X-ray fluorescence (pXRF) system

Tommi E. Tiihonen[1], Tuomo J. Nissinen[2], Petri A. Turhanen[3], Jouko J. Vepsäläinen[3], Joakim Riikonen[1], Vesa-Pekka Lehto[1,*]

[1]Department of Applied Physics, University of Eastern Finland, Yliopistonranta 1, FI-70211, Kuopio, Finland
[2]3AWater Oy, Microkatu 1, FI-70210, Kuopio, Finland
[3]School of Pharmacy, University of Eastern Finland, Yliopistonranta 1, FI-70211, Kuopio, Finland

**E-mail: vesa-pekka.lehto@uef.fi*

Table of Contents

Table S1. List of different approaches to preconcentration of the metals from aqueous solutions for determination of metal content with portable XRF from literature compared to the system developed in this study..................**S-3**

Figure S1. XRF spectra measured from MCF with adsorbed metals compared to blank measurement. **S-4**

Table S2. XRF emission lines used for analysis. **S-5**

Figure S2. The particle size distribution of the BP-TCPSi particles. **S-5**

Figure S3. Thermogravimetric analysis of TCPSi and BP-TCPSi samples. **S-6**

Figure S4. N_2 sorption isotherms measured from a) BP-TCPSi particles and b) the same particles with cross-linked PAA and CMC binders. **S-6**

Figure S5. Comparison of XRF intensities from the wet metal collecting filters used to calibrate the system right after the filtration and after drying the filters. **S-7**

Figure S6. The XRF intensities measured from the metal collecting filters after filtrating the water samples as a function of metal concentrations measured with ICP-MS from the initial water samples (linear axis). **S-7**

Figure S7. The XRF intensities measured from the metal collecting filters after filtrating the water samples as a function of metal concentrations measured with ICP-MS from the initial water samples (logarithmic axis)........**S-8**

Figure S8. Adsorption efficiencies of the metal collecting filters determined from the water samples used for calibration...**S-8**

Effect of pH, magnesium, and sodium content in water matrix on the adsorption of metals on the MCF **S-9**

Figure S9. Manganese S-9

Figure S10. Nickel S-9

Figure S11. Copper S-10

Figure S12. Zinc S-10

Figure S13. Lead S-11

Figure S14. Uranium S-11

Table S3. Temperature, electrical conductivity, pH, and metal concentrations of the water samples collected during the field measurements. **S-12**

References **S-12**

Table S1. List of different approaches to preconcentration of the metals from aqueous solutions for determination of metal content with portable XRF from literature compared to the system developed in this study.

Ref.	Metals	Preconcentration method	pH	Water volume (mL)	Detection limit (mg/L)*	Time (min)**
**This work**	Mn, Ni, Cu, Zn, Pb, U	Adsorption on BP-TCPSi filter using flow-through setup	5.0 - 8.5	10	0.05 – 0.15	< 15
1	Fe, Cu, Zn, Se, Pb	APDC precipitation and filtration	4.0 (adjusted)	300	0.002 – 0.006	< 30
2	As, Bi, Se, Cd, Co, Cu, Fe, Ni, Pb, V, Zn	PDTC compound precipitation and filtration through paraffin-treated cellulose filter	2 M HCl for As, Bi, Se. pH 4.8 - 5.2 for the rest.	100	0.001 – 0.040	< 30
3	As, Se, Cr	Filtration through Ti-loaded anion-exchange disk	4.0 (adjusted)	50	0.001	< 15
4	Ni, Cu, Zn, Pb, Cd	Filtration through 3 M Empore chelating disks	6 – 7	1000	0.001 – 0.007	< 30
5	Cd, Pb	Filtration through TiO_2 immobilized on cellulose filter paper	8	1000	< 0.001	> 180
6	V, Cr, Mn, Fe, Co, Ni, Cu, Zn	Drying of water droplet on a solid support	-	0.2	2 – 18	< 30
7	Pb	Filtration through commercial activated carbon felt	> 5	2000	0.001	< 45

***Depends on the measured metal and water matrix.**
****Time taken from sampling the water to acquiring the results.**

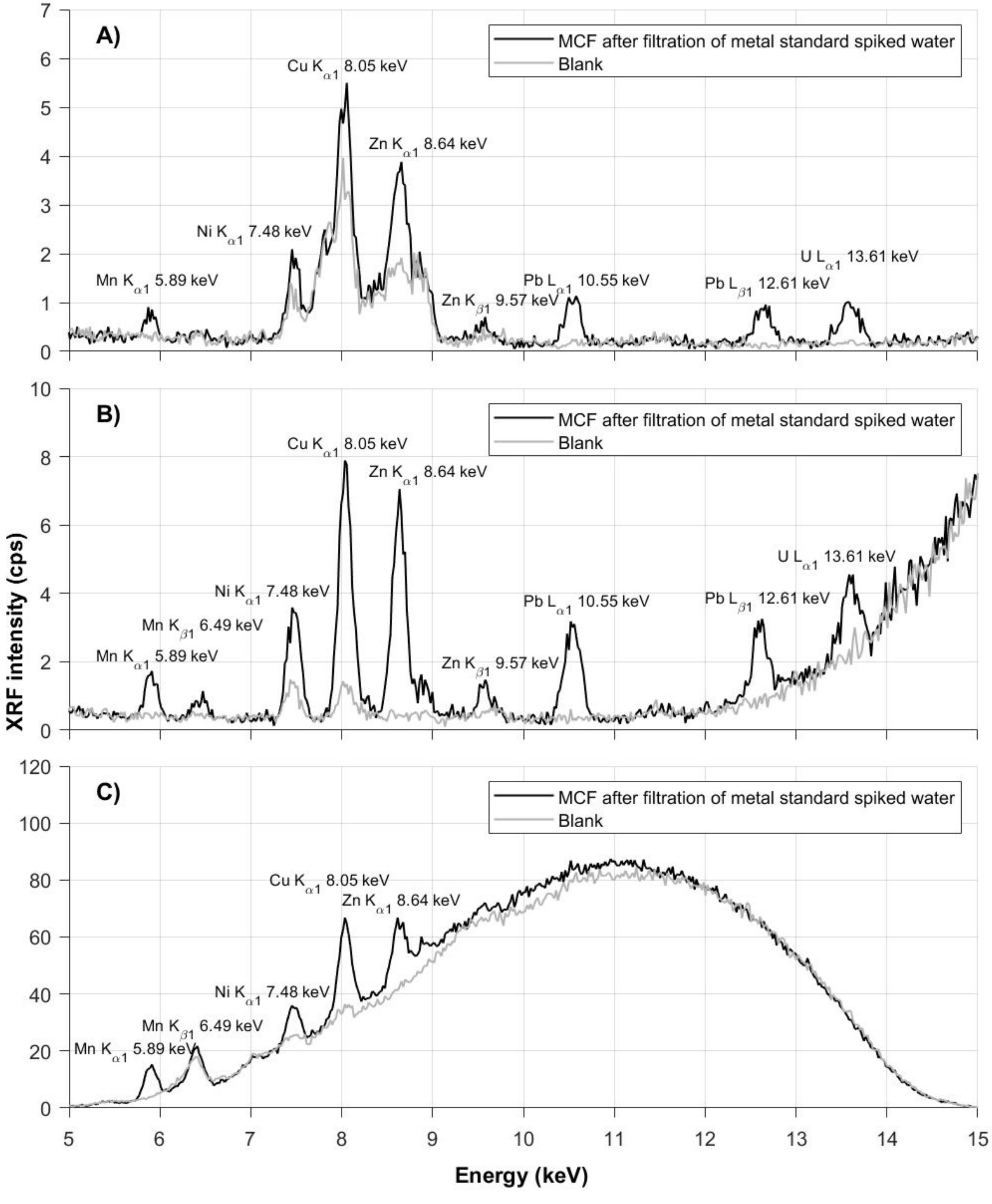

Figure S1. Measured XRF spectra from the MCF with 5.1 µg of Mn, 3.9 µg of Ni, 7.9 µg of Cu, 6.9 µg of Zn, 4.1 µg of Pb and 3.6 µg of U adsorbed from 10 mL water sample. Blank measurement from MCF with no adsorbed metals is provided for comparison. Characteristic emission lines of the metals are marked in the spectra measured with the parameters A) V = 40 kV, A = 80 μA, Filter 3, B) V= 40 kV, A = 83 μA, Filter 1 and C) V = 15 kV, A = 125 μA, Filter 5 using DPO-2000C XRF analyzer.

Table S2. The XRF spectra were analyzed with a computer to determine the metal content of the MCF from the characteristic XRF emission lines of Mn, Ni, Cu, Zn, Pb and U. The sum of XRF counts were calculated within Regions of Interests (ROIs).

Metal	XRF emission line	Emission energy (keV)	ROI (keV)
**Mn**	Ka_1	5.89	5.70 – 6.10
**Ni**	Ka_1	7.48	7.25 – 7.70
**Cu**	Ka_1	8.05	7.82 – 8.28
**Zn**	Ka_1	8.64	8.40 – 8.88
**Pb**	La_1	10.55	10.30 – 10.80
**U**	La_1	13.61	13.30 – 13.90

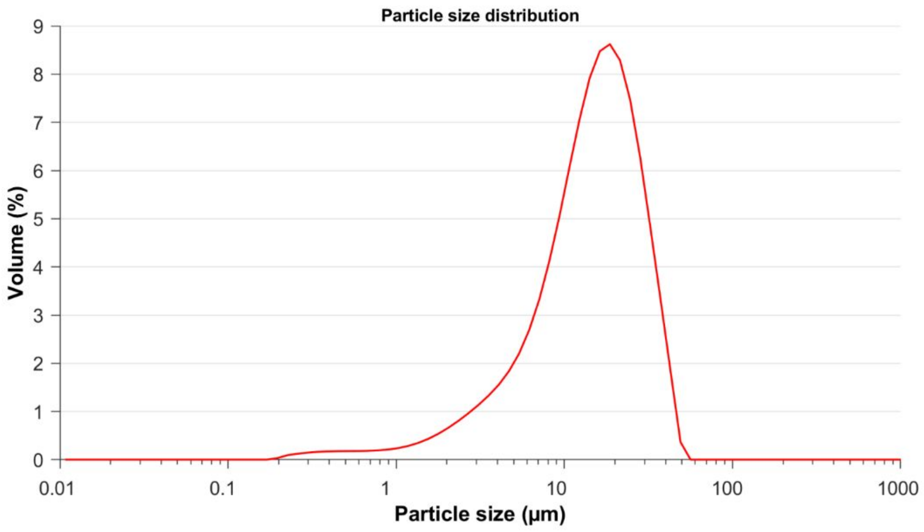

Figure S2. The particle size distribution of the BP-TCPSi particle

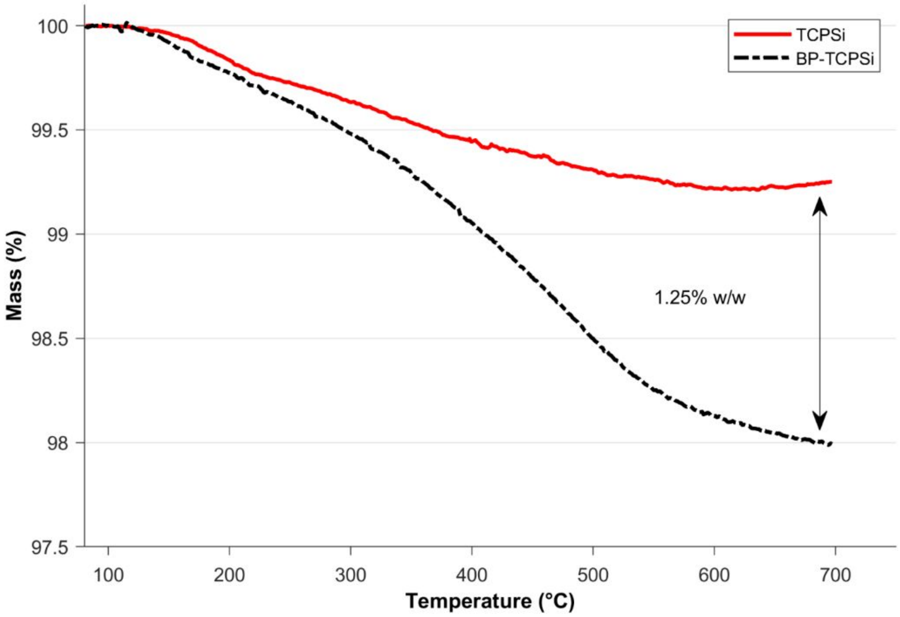

Figure S3. Thermogravimetric analysis of TCPSi and BP-TCPSi samples. The BP content of BP-TCPSi was determined from the difference in the mass loss between the samples.

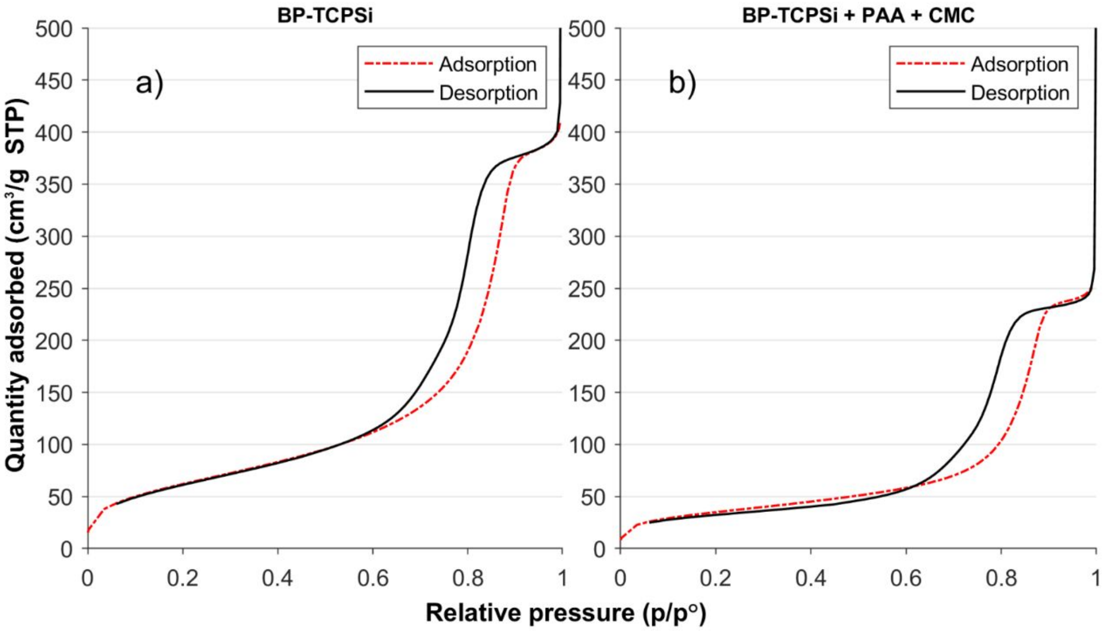

Figure S4. N_2 sorption isotherms measured from a) BP-TCPSi particles and b) the same particles with cross-linked PAA and CMC binders.

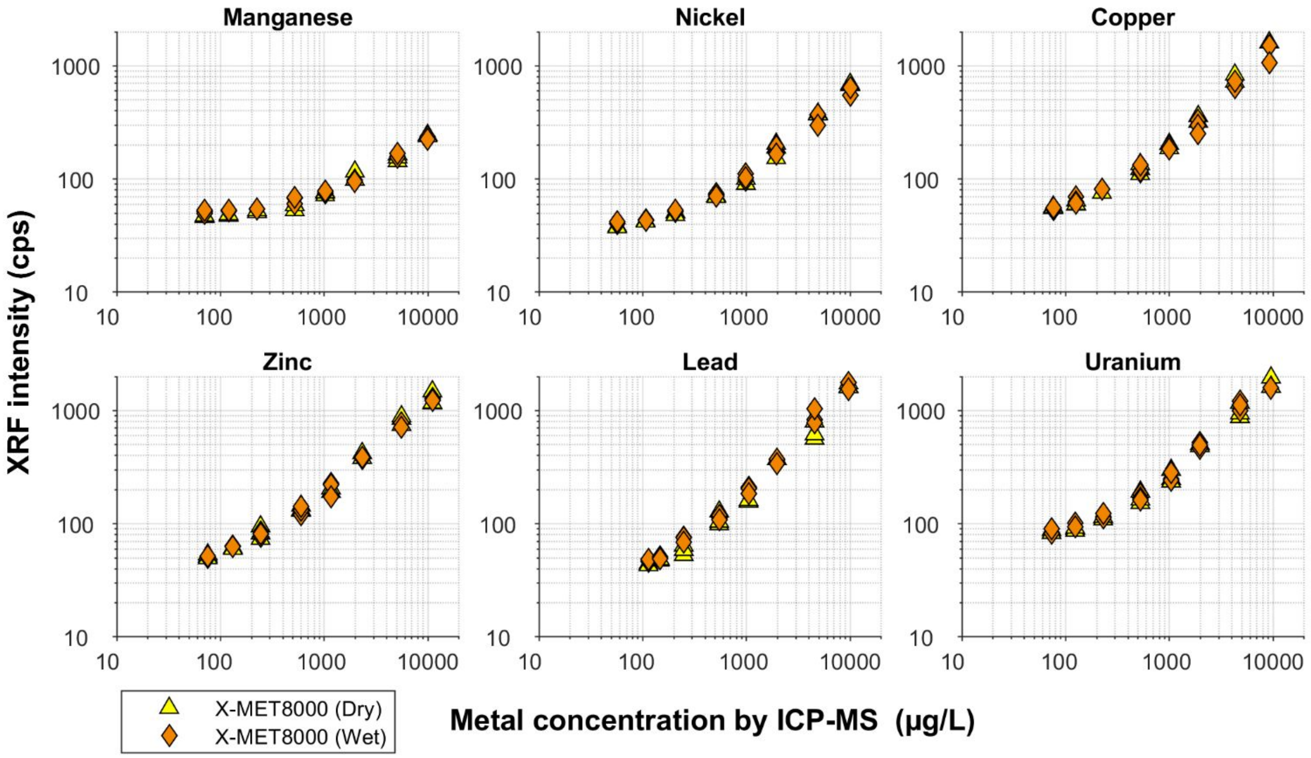

Figure S5. Comparison of XRF intensities from the wet metal collecting filters used to calibrate the system right after the filtration and after drying the filters

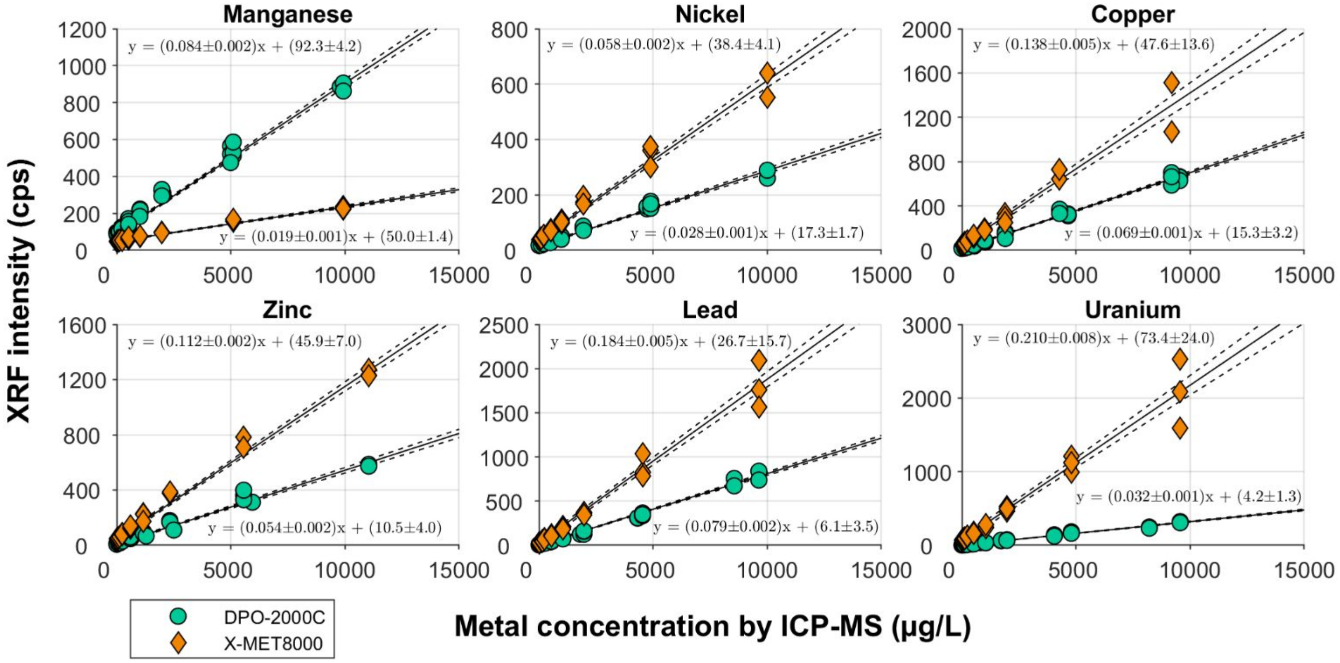

Figure S6. The XRF intensities measured from the metal collecting filters after filtrating the water samples as a function of metal concentrations measured with ICP-MS from the initial water samples (linear axis). 95% confidence bands of the fitted lines are shown by the dashed lines.

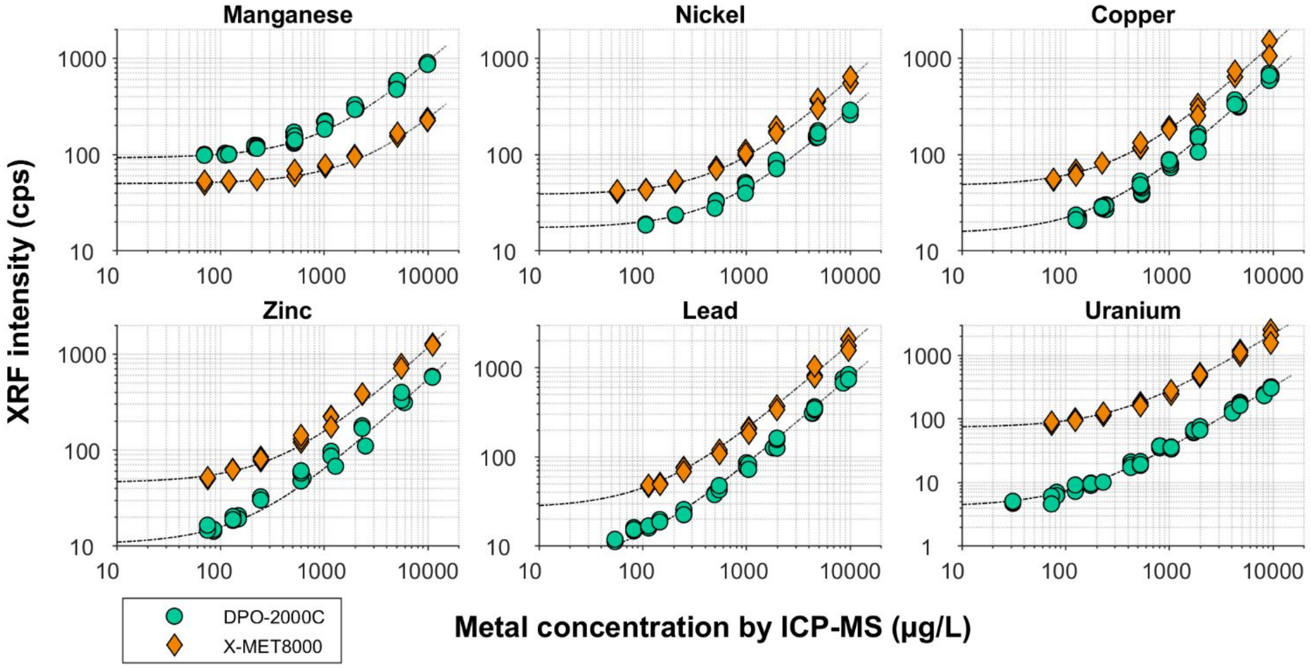

Figure S7. The XRF intensities measured from the metal collecting filters after filtrating the water samples as a function of metal concentrations measured with ICP-MS from the initial water samples (logarithmic axis).

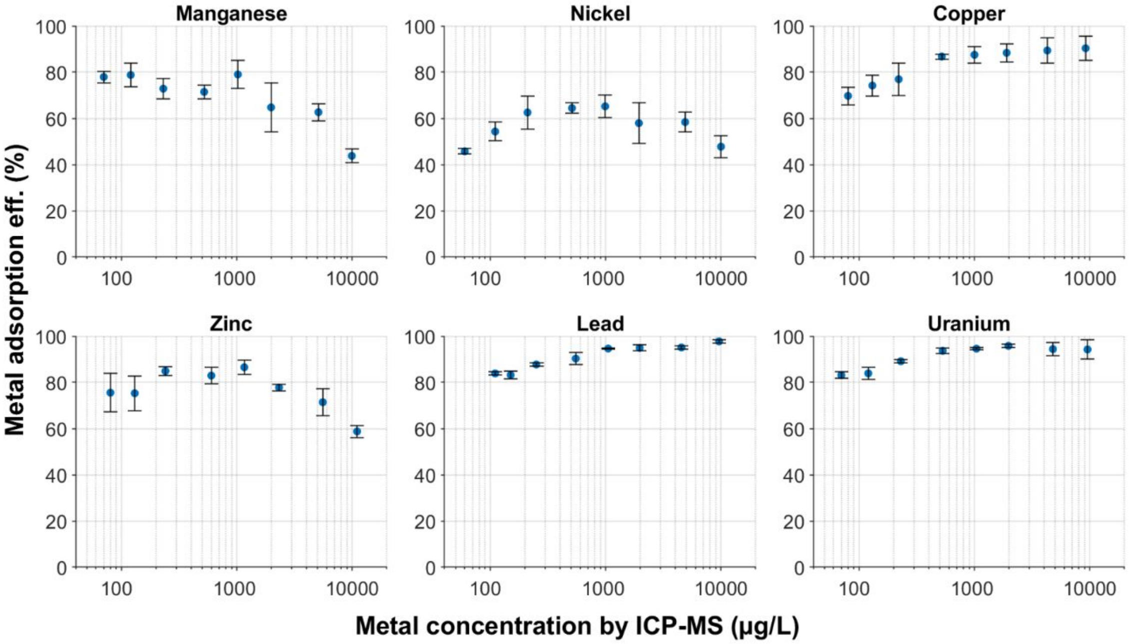

Figure S8. Adsorption efficiencies of the metal collecting filters determined from the water samples used for calibration. The samples were prepared by spiking lake water with one of the metals and adjusting the pH to 7 with NaOH.

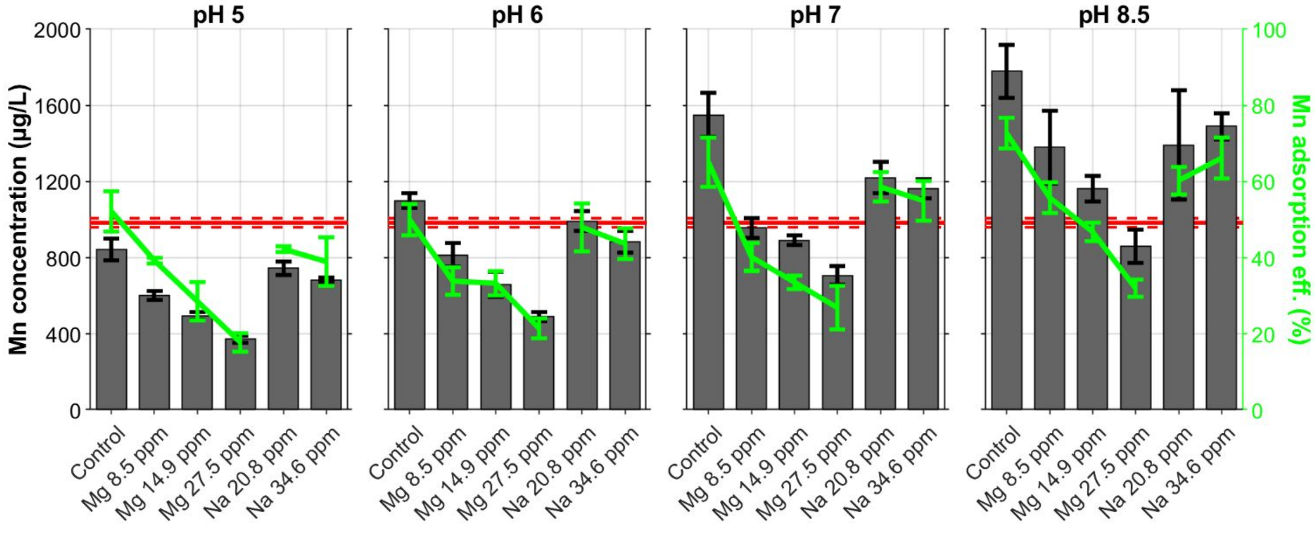

Figure S9. Manganese concentrations measured from lake water matrices containing different concentrations of magnesium and sodium in pH 5, 6, 7 and 8.5. The control sample contains 2.5 mg/L of magnesium and 7.2 mg/L of sodium similar to the water samples used in the calibration of the pXRF system. Bar chart shows the manganese concentrations reported by the pXRF system. The horizontal solid line is the average metal concentration of the water samples measured with ICP-MS and the dashed lines represent the standard deviation. Corresponding adsorption efficiencies of the metal are shown on the right vertical axis.

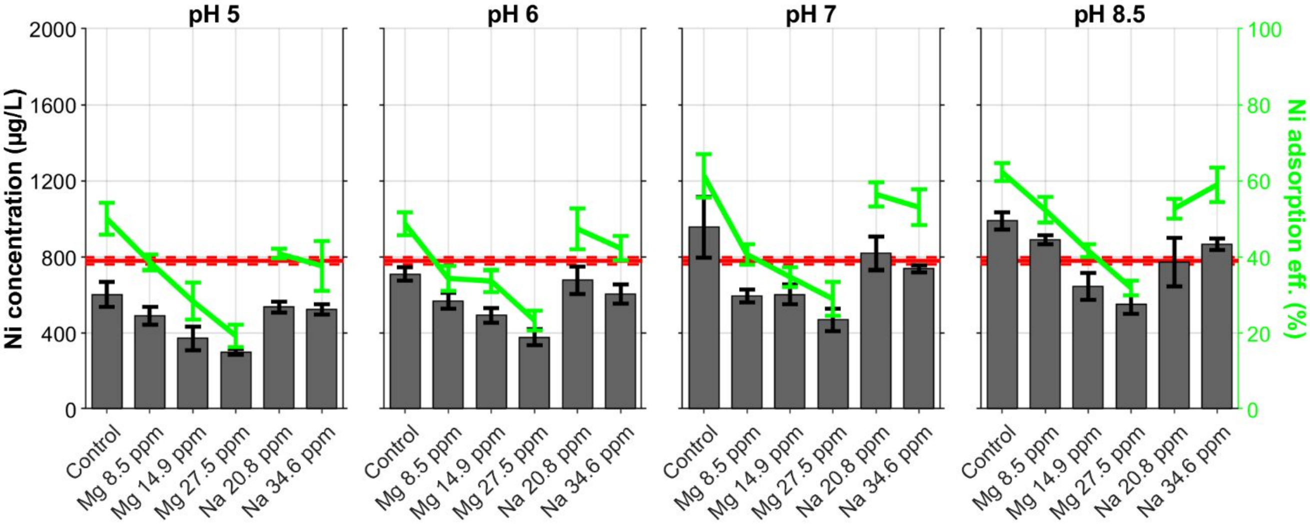

Figure S10. Nickel concentrations measured from lake water matrices containing different concentrations of magnesium and sodium in pH 5, 6, 7 and 8.5. The control sample contains 2.5 mg/L of magnesium and 7.2 mg/L of sodium similar to the water samples used in the calibration of the pXRF system. Bar chart shows the manganese concentrations reported by the pXRF system. The horizontal solid line is the average metal concentration of the water samples measured with ICP-MS and the dashed lines represent the standard deviation. Corresponding adsorption efficiencies of the metal are shown on the right vertical axis.

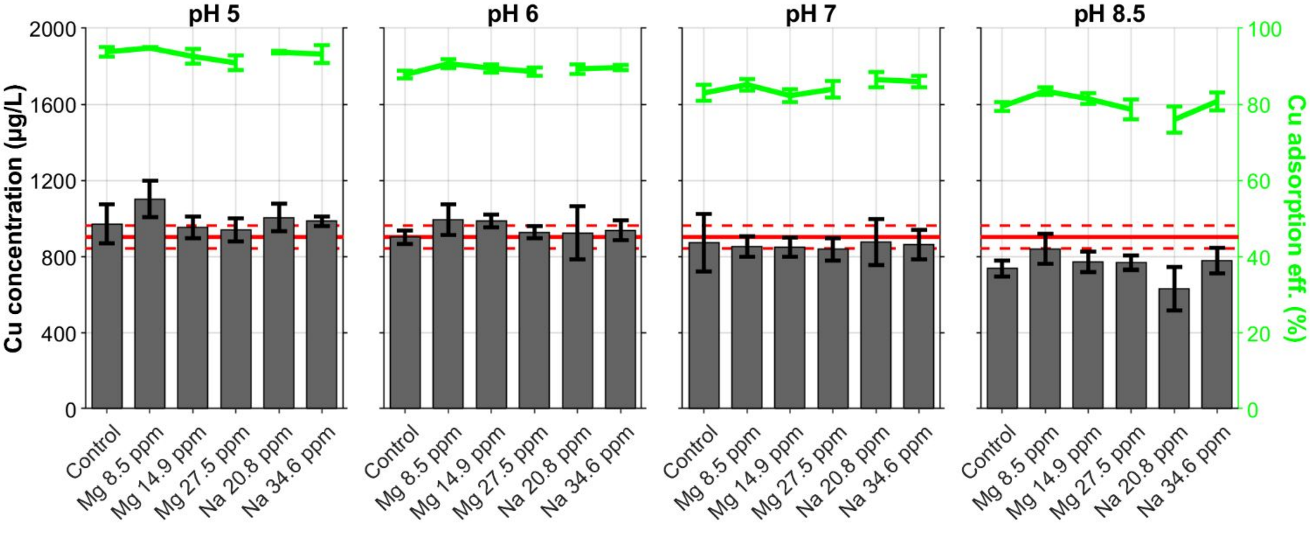

Figure S11. Copper concentrations measured from lake water matrices containing different concentrations of magnesium and sodium in pH 5, 6, 7 and 8.5. The control sample contains 2.5 mg/L of magnesium and 7.2 mg/L of sodium similar to the water samples used in the calibration of the pXRF system. Bar chart shows the manganese concentrations reported by the pXRF system. The horizontal solid line is

the average metal concentration of the water samples measured with ICP-MS and the dashed lines represent the standard deviation. Corresponding adsorption efficiencies of the metal are shown on the right vertical axis.

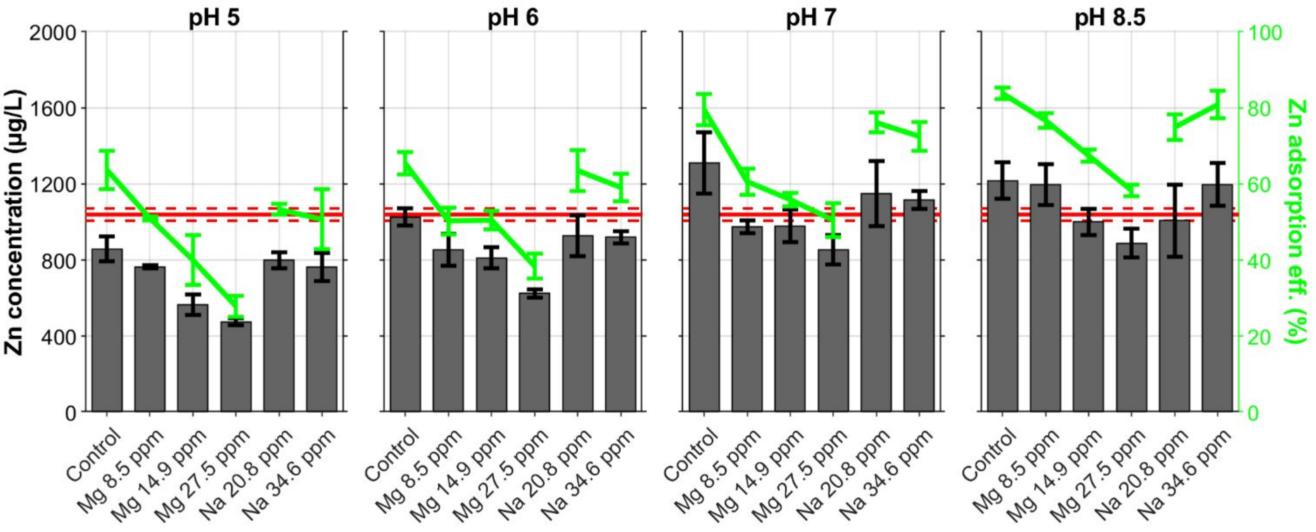

Figure S12. Zinc concentrations measured from lake water matrices containing different concentrations of magnesium and sodium in pH 5, 6, 7 and 8.5. The control sample contains 2.5 mg/L of magnesium and 7.2 mg/L of sodium similar to the water samples used in the calibration of the pXRF system. Bar chart shows the manganese concentrations reported by the pXRF system. The horizontal solid line is the average metal concentration of the water samples measured with ICP-MS and the dashed lines represent the standard deviation. Corresponding adsorption efficiencies of the metal are shown on the right vertical axis.

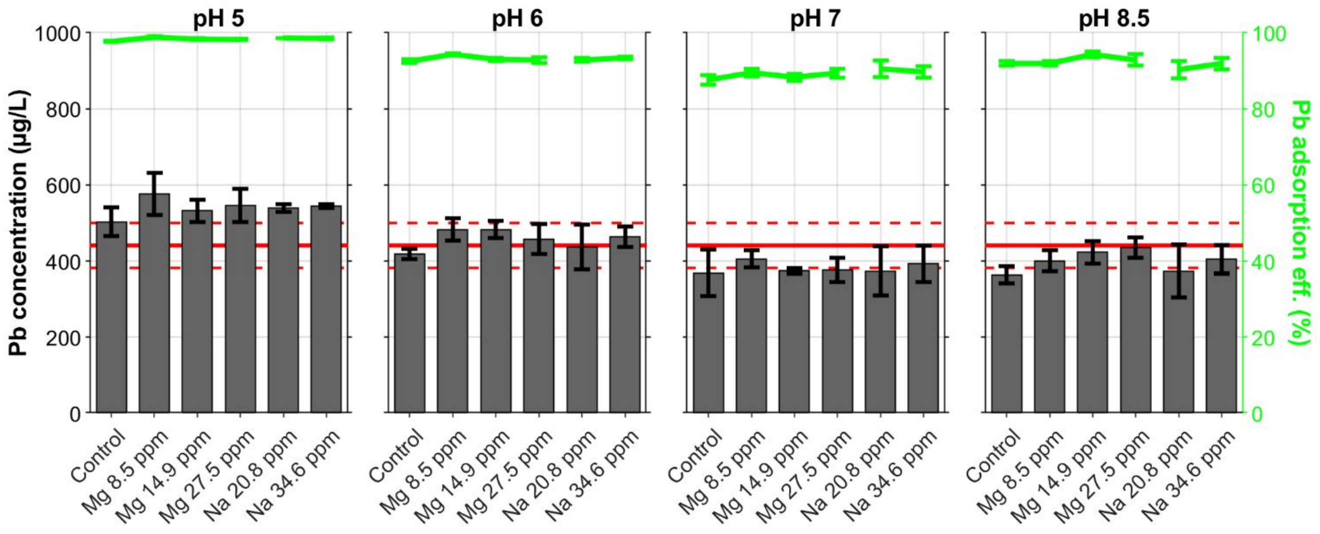

Figure S13. Lead concentrations measured from lake water matrices containing different concentrations of magnesium and sodium in pH 5, 6, 7 and 8.5. The control sample contains 2.5 mg/L of magnesium and 7.2 mg/L of sodium similar to the water samples used in the calibration of the pXRF system. Bar chart shows the manganese concentrations reported by the pXRF system. The horizontal solid line is the average metal concentration of the water samples measured with ICP-MS and the dashed lines represent the standard deviation. Corresponding adsorption efficiencies of the metal are shown on the right vertical axis.

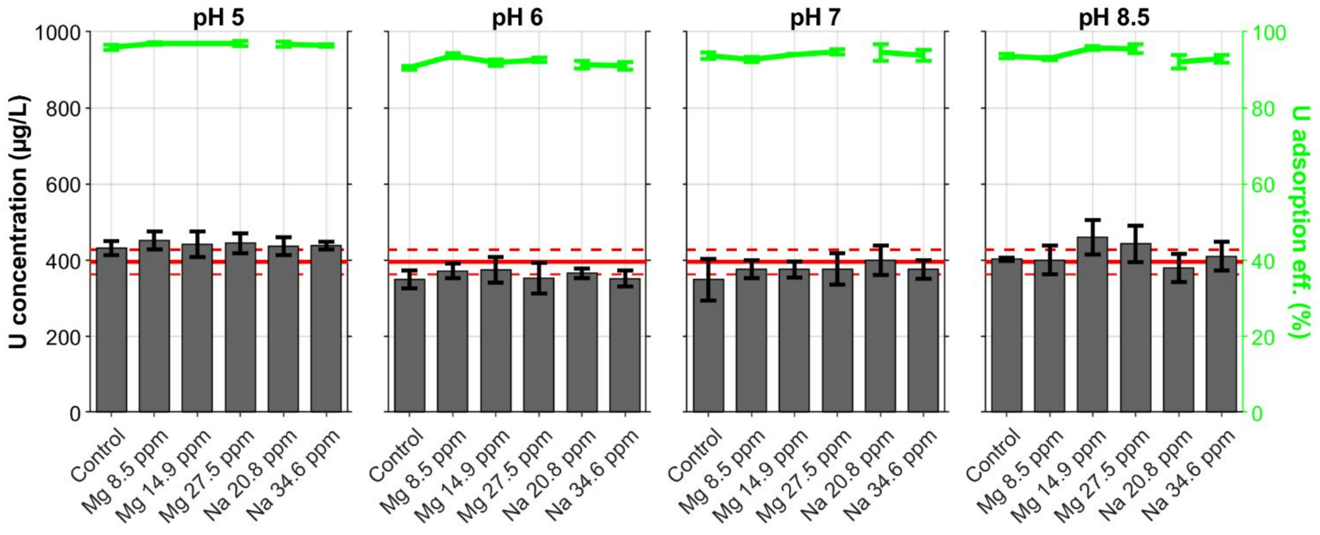

Figure S14. Uranium concentrations measured from lake water matrices containing different concentrations of magnesium and sodium in pH 5, 6, 7 and 8.5. The control sample contains 2.5 mg/L of magnesium and 7.2 mg/L of sodium similar to the water samples used in the calibration of the pXRF system. Bar chart shows the manganese concentrations reported by the pXRF system. The horizontal solid line is the average metal concentration of the water samples measured with ICP-MS and the dashed lines represent the standard deviation. Corresponding adsorption efficiencies of the metal are shown on the right vertical axis.

Table S3. Temperature, electrical conductivity, pH, and metal concentrations of the water samples (S1-S16, n=1) collected during the field measurements. In samples S12 - S16 all metal concentrations were below the detection limits of the pXRF system.

Sample	T (°C)	EC (µS/cm)	pH	Mn (µg/L)		Ni (µg/L)		Cu (µg/L)		Zn (µg/L)	
				ICP-MS	DPO 2000C	ICP-MS	DPO 2000C	ICP-MS	DPO 2000C	ICP-MS	DPO 2000C
**S1**	16.4	692	6.7	4590	3569	6	< LOD	4	< LOD	6	< LOD
**S2**	16.4	367	6.8	564	540	4	< LOD	26	< LOD	30	41
**S3**	16.4	515	6.8	590	582	2	< LOD	1	< LOD	<5	< LOD
**S4**	16.4	565	6.6	3510	2847	4	< LOD	0	< LOD	<5	< LOD
**S5**	16.4	529	6.3	215	207	9	< LOD	4	< LOD	5	< LOD
**S6**	16.4	495	6.0	1790	1534	15	< LOD	3	< LOD	64	70
**S7**	16.4	562	6.5	2	< LOD	640	422	172	132	1650	1645
**S8**	17.6	382	6.7	4	< LOD	7	< LOD	5	< LOD	23	< LOD
**S9**	16.4	529	6.5	797	756	7	< LOD	1	< LOD	<5	< LOD
**S10**	16.3	583	6.5	200	128	7	< LOD	0	< LOD	<5	< LOD
**S11**	12.9	486	6.5	584	223	2350	1326	2220	3547	2130	1699
**S12**	14.3	285	6.1	3	< LOD	2	< LOD	0	< LOD	6	< LOD
**S13**	12.9	232	5.7	4	< LOD	7	< LOD	20	< LOD	9	< LOD
**S14**	11.9	225	5.6	6	< LOD	2	< LOD	26	< LOD	<5	< LOD
**S15**	6.7	138	7.8	16	< LOD	2	< LOD	3	< LOD	<5	< LOD
**S16**	7.5	99	7.3	15	< LOD	2	< LOD	2	< LOD	<5	< LOD

References

1. Melquiades, F. L.; Parreira, P. S.; Yabe, M. J.; Corazza, M. Z.; Funfas, R.; Appoloni, C. R. Factorial Design for Fe, Cu, Zn, Se and Pb Preconcentration Optimization with APDC and Analysis with a Portable X-Ray Fluorescence System. *Talanta* **2007**, *73*, 121-126.

2. Gordeeva, V. P.; Statkus, M. A.; Tsysin, G. I.; Zolotov, Y. A. X-Ray Fluorescence Determination of as, Bi, Co, Cu, Fe, Ni, Pb, Se, V and Zn in Natural Water and Soil Extracts After Preconcentration of their Pyrrolidinedithiocarbamates on Cellulose Filters. *Talanta* **2003**, *61*, 315-329.

3. Hagiwara, K.; Koike, Y.; Aizawa, M.; Nakamura, T. On-Site Determination of Arsenic, Selenium, and Chromium(VI) in Drinking Water using a Solid-Phase Extraction Disk/Handheld X-Ray Fluorescence Spectrometer. *Anal. Sci.* **2018**, *34*, 1309-1315.

4. Margui, E.; Hidalgo, M.; Queralt, I.; Van Meel, K.; Fontas, C. Analytical Capabilities of Laboratory, Benchtop and Handheld X-Ray Fluorescence Systems for Detection of Metals in Aqueous Samples Pre-Concentrated with Solid-Phase Extraction Disks. *Spectrochim. Acta B* **2012**, *67*, 17-23.

5. Lin, X.; Li, S.; Zheng, F. An Integrated System for Field Analysis of Cd(II) and Pb(II) Via Preconcentration using Nano-TiO2/Cellulose Paper Composite and Subsequent Detection with a Portable X-Ray Fluorescence Spectrometer. *RSC Adv.* **2016**, *6*, 9002-9006.

6. Pessanha, S.; Margui, E.; Carvalho, M. L.; Queralt, I. A Simple and Sustainable Portable Triaxial Energy Dispersive X-Ray Fluorescence Method for in Situ Multielemental Analysis of Mining Water Samples. *Spectrochim. Acta B: At. Spectrosc.* **2020**, *164*.

7. Tighe, M.; Bielski, M.; Wilson, M.; Ruscio-Atkinson, G.; Peaslee, G. F.; Lieberman, M. A Sensitive XRF Screening Method for Lead in Drinking Water. *Anal. Chem.* **2020**, *92*, 4949-4953.
